# Standard or Fin SIGN® nail? which option is better for the treatment of femoral fractures in low and middle-income countries?

**DOI:** 10.1007/s00264-024-06192-7

**Published:** 2024-05-18

**Authors:** Juan Carlos Perdomo-Lizarraga, Dennys J. Andrade-Arellano, Marco Necchi, Marcello Zavatta, Marcella Ryan-Coker, Richmond Dixon-Cole, Ernesto Muñoz-Mahamud, Andrés Combalia

**Affiliations:** 1 Emergency Surgery Centre, Goderich-Freetown, Sierra Leone; 2 Department of Orthopedics, University Hospital of Jaen, 23009 Jaen City, Spain; 3 Orthopaedic Department, IRCCS Galeazzi Hospital- Sant’Ambrogio, 20157 Milan, Italy; 4 Hand Surgery Department, MultiMedica Hospital, 21053 Castellanza, Italy; 5 Medical Division of Emergency NGO, Milan, Italy; 6 Department of Surgery, University of Nairobi, Nairobi, Kenya; 7 College of Medicine and Veterinary Medicine, University of Edinburgh, Edinburgh, UK; 8 Departament de Cirurgia i Especialitats Medicoquirúrgiques, Facultat de Medicina i Ciències de La SalutUniversitat de Barcelona (UB), c. Casanova, 143, 08036 Barcelona, Spain; 9 Institut d’Investigacions Biomèdiques August Pi i Sunyer (IDIBAPS), C. Villarroel, 170, 08036 Barcelona, Spain; 10 Facultat de Medicina i Ciències de La Salut, Universitat de Barcelona (UB), c. Casanova, 143, 08036 Barcelona, Spain

**Keywords:** Femoral fractures, Orthopedic surgery, Intramedullary fracture fixation, Developing countries

## Abstract

**Purpose:**

Femoral fractures are common in low and middle-income countries (LMIC), predominantly caused by high-energy trauma. The surgical implant generation network (SIGN®) program offers two different intramedullary nails in LMIC which are designed to be used without image intensifier free of charge for the patients: the SIGN standard nail (SSN®) and the SIGN Fin nail (SFN®). This study aimed to compare the results of the SSN® and the SFN® for the treatment of middle and distal shaft femoral fractures through a retrograde approach.

**Material and Methods:**

This was a retrospective, descriptive, and non-experimental study including all consecutive patients who underwent surgical management of middle or distal shaft femoral fracture between January 2017 and May 2022 in an NGO hospital located in Freetown, Sierra Leone. The duration of surgery, type of reduction, complications like screw loosening, implant migration, anterior knee pain and non-union rate at six months of follow up were evaluated.

**Results:**

A total of 122 patients were included in the study. Group A: 60 patients were managed with SSN® and Group B: 62 patients with SFN®. The mean operative time was 104 min with SSN® and 78 with SFN® (*p* < 0.001). Open reduction of the fracture was necessary in ten (16.7%) patients with SSN® and 12 (19.4%) patients treated with SFN® (*p* = 0.69). Non-union was observed in one (1.7%) patient with SSN® and two (3.2%) patients with SFN® (*p* = 0.57).

**Conclusions:**

Both options seem equally effective in treating midshaft and distal femoral shaft fractures. The SFN® reduces the surgical time, due to this fact, in polytraumatized patients, patients with bilateral femur fracture or patients with ipsilateral tibia fracture, it can be considered as the best option to be used. There was no statistical difference in the complications presented by the two groups.

## Introduction

Femoral shaft fractures correspond to approximately 17% of all musculoskeletal injuries in low and middle-income countries (LMIC) [1], and distal femoral fractures account for 3–6% of all femoral fractures [2]. The 80–90% of the world's trauma occurs in LMIC, mainly affecting the working population of these countries [3]. Sierra Leone, a low-income West African country is no exception; the country has a significant trauma burden, primarily due to road traffic accidents (RTA) [4]. It has been reported that 40% of femoral fractures are associated with RTA and high energy trauma [5].

As a matter of fact, Sierra Leona’s trauma burden exceeds its orthopaedic and trauma capacity [6]; this could be explained because the main limitations for orthopaedics in Africa are the lack of trained personnel, lack of supplies, lack of budget, migration of orthopaedic surgeons to other countries with better opportunities [7].

In many LMIC, hospital resources are sometimes dependent on non-governmental and humanitarian organizations (NGO) and the most commonly used or available osteosynthesis materials in low-resourced settings include external fixators, conventional non-locking plates and intramedullary nails [8, 9].

**Fig. 1:**
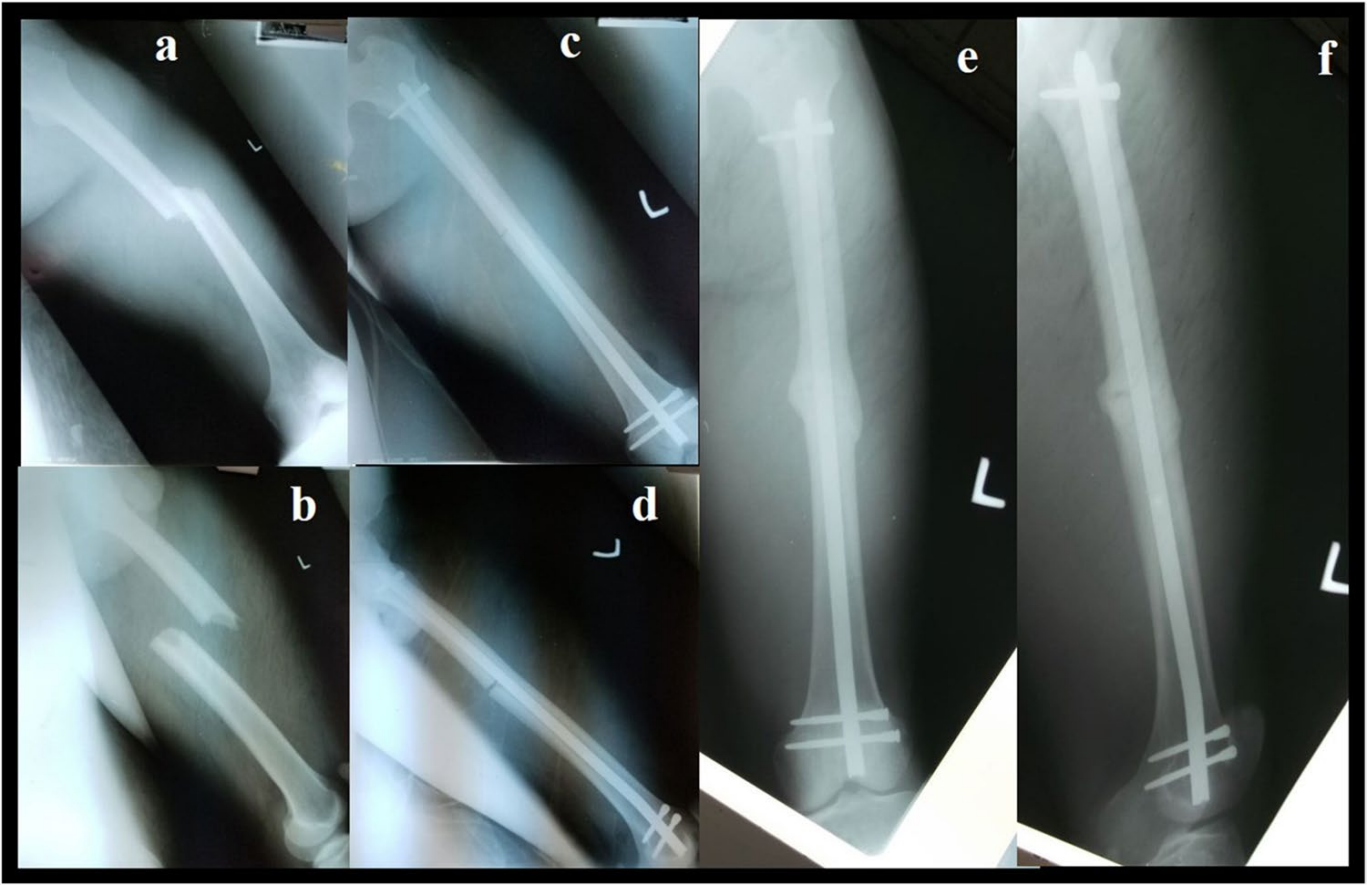
40 year old male patient with femoral midshaft fractures treated using SSN®, (**a**, **b**) preoperative radiographs, (**c**, **d**) postoperative radiographs, (**e**, **f**) consolidation at the follow up at 12 months

**Fig. 2:**
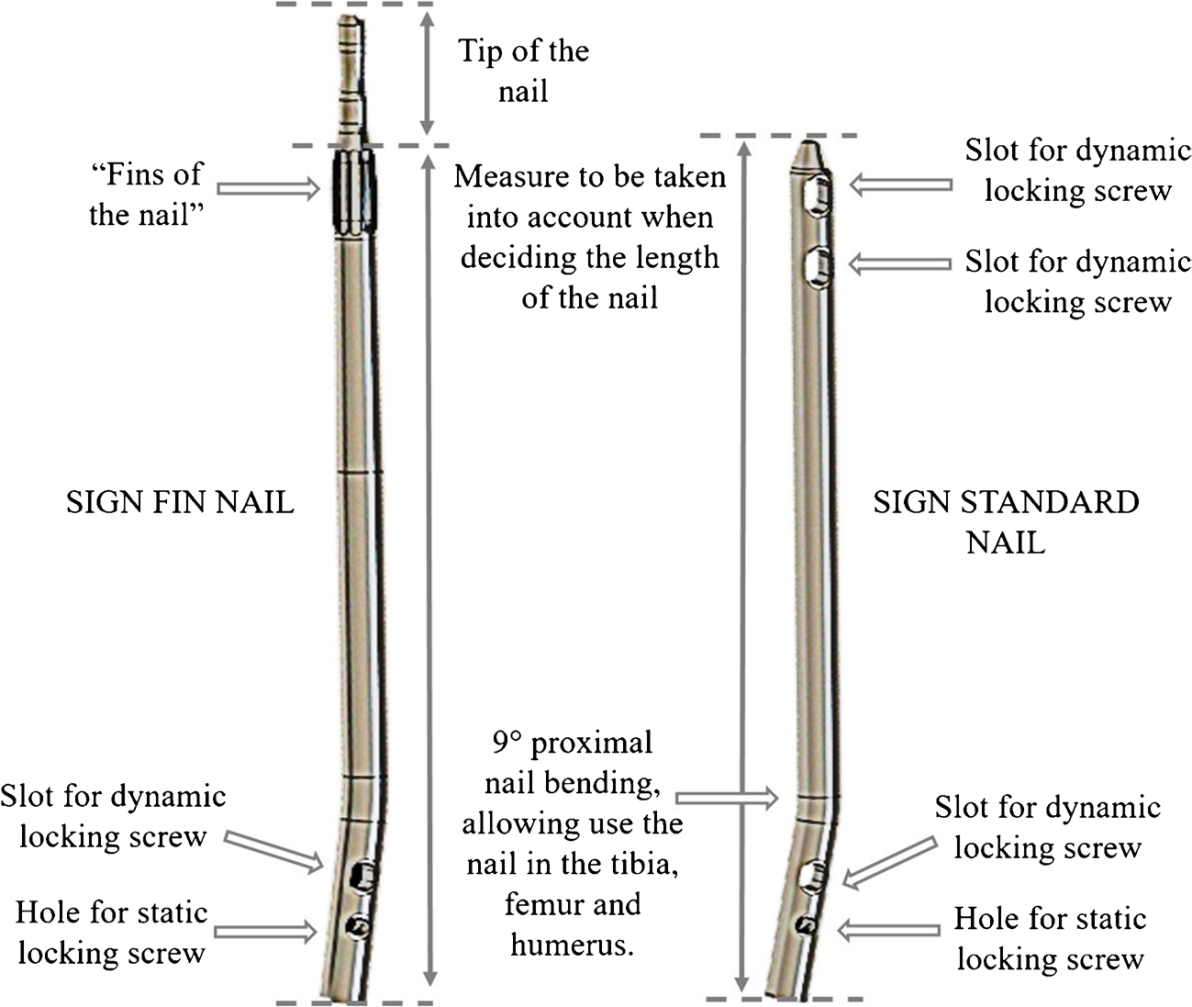
Characteristics of SIGN® Standard Nail and SIGN® Fin Nail

The availability of surgical implant generation network® (SIGN®) intramedullary nails has revolutionized fracture management in some low-resourced countries [10, 11]. SIGN® is a registered non-profit corporation based in Washington State, in the United States of America [12], which, through donations, develops and distributes implants designed for use in LIMC where resources, including image intensifiers and fracture tables, are limited. For femoral retrograde approaches (Fig. 1) the SIGN® program offers two different intramedullary nails: the SIGN standard nail (SSN®) and the SIGN Fin nail (SFN®) [13], the SFN® has a system of wider “fins” (Fig. 2) that fit into the medullary canal locking the nail and preventing rotational movements [14].

The purpose of this study was to compare the results in terms of the duration of surgery and the type of reduction performed in patients treated using SSN® with those managed with SFN® for the treatment of middle and distal shaft femoral fractures using a retrograde nailing approach.

As secondary objectives, we also analyzed the main mechanisms of injury, fracture patterns according to the AO classification [15]. Open fractures were classified according to the Gustilo-Anderson classification [16]. Other parameters that we evaluated were the complications of the patients at six months of follow-up: anterior knee pain during full weight bearing, non-union, screw loosening and nail migration.

## Materials and methods

### Study setting

This study was developed is an NGO hospital located in Goderich, Freetown, Sierra Leone, active since 2001 where all the patients are treated free of charge, the hospital is equipped with 85 beds, divided in an intensive care unit with eight beds, an operating theatre with three operating rooms, six wards, an outpatient department, a dressing room, an X-ray room, a physiotherapy room, and a casting room [8, 9].

All patients in this study were operated by surgeons with more than three years of experience: international staff (JCP, DA, MN) and two national physicians (MRC, RDC). In all cases, patients were placed in the supine decubitus position, a triangular support was used to achieve flexion on 30–40° of the knee and a transpatellar tendon approach was performed. The choice of nail was at the discretion of the surgeon at the time of the surgery; there were no specific criteria for deciding which nail to use. All surgeries were performed using the image intensifier.

### Study design

**Fig. 3:**
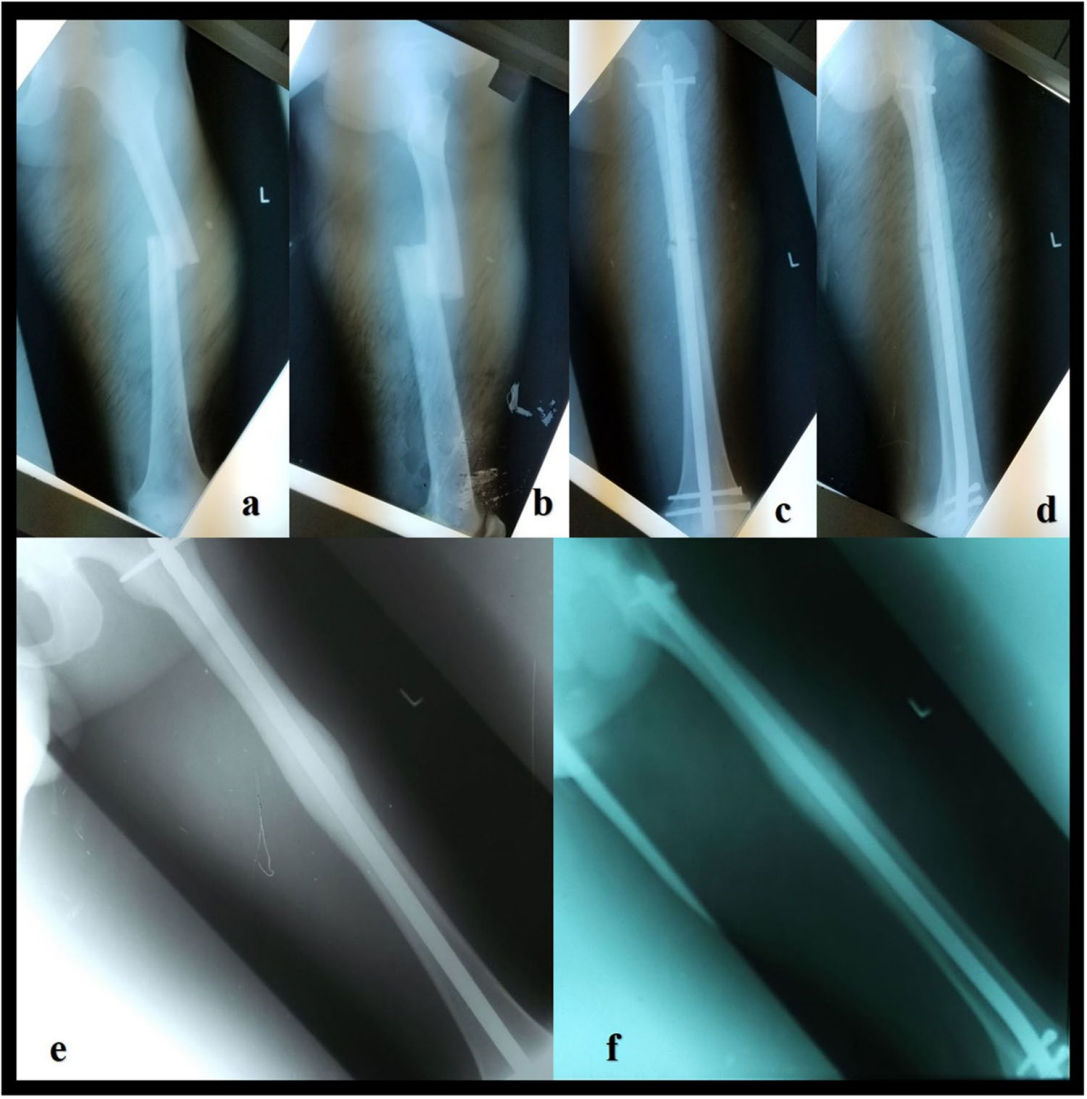
A 38 year old male patient with femoral midshaft fractures treated using SSN®, (**a**, **b**) preoperative radiographs, (**c**, **d**) postoperative radiographs, (**e**, **f**) consolidation at the follow up at 12 months

This was a retrospective, descriptive, and non-experimental study including all consecutive patients who underwent surgical management of middle or distal shaft femoral fracture between January 2017 and May 2022 in Freetown, Sierra Leone. The inclusion criteria were patients who underwent surgical interventions within 90 days post-injury using the SSN® or SFN® system in a retrograde femoral nailing approach (Fig. 3). The patients were divided into two groups: Group A patients were managed with SIGN® Standard Nail and Group B patients with SIGN® Fin Nail (Table 1).

**Table 1:** Patients demographics and injury characteristics

VARIABLE	STANDARD NAIL *n*=60		FIN NAIL *n*=62		*P* value
	*n*	SD	*n*	SD	
Mean age	36.5	14.5	33.6	13.6	0.25
VARIABLE	STANDARD NAIL *n*=60		FIN NAIL *n*=62		*P* value
	*n*	%	*n*	%	
Female	16	26.7	25	40.3	0.11
Male	44	73.3	37	59.7	
Mechanism of injury:					
Road traffic accidents	52	86.7	60	96.8	**0.04**
Fall	7	11.7	2	3.2	0.07
Gunshot	1	1.6	0	0	0.30
Extremity:					
Right	34	56.7	34	54.8	0.83
Left	26	43.3	28	45.2	
Other injuries:					
No	27	45	16	25.8	**0.02**
Ipsilateral tibia	5	8.3	9	14.5	0.28
Contralateral tibia	1	1.6	1	1.6	0.98
Contralateral femur	8	13.3	5	8.1	0.34
Pelvis	2	3.3	1	1.6	0.53
Superior limb fractures	1	1.6	2	3.2	0.57
Soft tissue wounds	2	3.3	3	4.8	0.67
Mandible fracture	1	1.6	0	0	0.30
More of 3 diagnoses	12	20	25	40.3	**0.01**

The follow-up of the patients was done in the outpatient department, at six weeks, 12 weeks, and six months. At each of the follow-up visits, patients were evaluated to rule out clinical signs of postoperative infection, deformities, anterior knee pain at full weight bearing and range of joint mobility greater than 90 degrees, X-rays were also taken to determine the presence of bone callus formation, to show complications such as screws loosening or migration of nails.

The assessment of the presence of callus was performed by two of the international orthopaedics staff (JCP,DA), who reviewed the patients' radiographs and used the modified radiographic union score for tibia (mRUST) [17, 18] to ensure uniformity between the two observers.

### Ethical requirements

On arrival to the hospital, each patient or immediate family member (in the case of children) signed an informed consent for admission to the hospital. The data used in the study were obtained from the medical records of the patients reflected in the SIGN® Online Surgical Database (SOSD). Data were collected retrospectively and analyzed pseudo-anonymously. This study was reviewed and approved by the independent Institutional Review Board of the NGO EMERGENCY.

### Statistical analysis

Continuous variables are described as mean and standard deviation (SD), and categorical variables are described as absolute numbers and percentages. Continuous variables were compared using Student's t-test and categorical variables using the Chi-square test. Data were collected in an Excel file, and analyses were performed using IBM SPSS 29.0.0 Statistical Package (SPSS, Inc., Chicago, IL).

## Results

One hundred twenty-two patients with femoral fractures treated with SSN or SFN were included in the analysis. Group A consisted of 60 (49.2%) patients (16 females and 44 males) and Group B consisted of 62 (50.8%) patients (25 females and 37 males). A mean age of 36.5 (SD 14.5) years in group A and 33.6 (SD 13.6) years in group B (*p* = 0.25).

The predominant mechanism of injury was RTA in 86.7% (52 of 60) patients in group A and 96.8% (60 of 62) of the patients in group B (*p* = 0.04). Twenty-seven (45%) patients in group A and 16 patients (25.8%) in group B were admitted with a unique diagnosis of femoral fracture (*p* = 0.02). Twenty-one patients from both groups were admitted with two diagnoses, the first diagnosis related to femur fracture and the second diagnosis was any other bone fracture, head injury, chest trauma or blunt abdominal trauma, representing 35% and 33.9% respectively (*p* = 0.89). The number of patients admitted with three or more diagnoses was 12 (20%) in group A and 25 (40.3%) in group B (*p* = 0.01).

Patients admitted with closed femoral fractures were 49 (81.7%) in group A and 47 (75.8%) in group B (*p* = 0.64). Open fractures occurred in 11 (18.3%) and 15 (24.2%) patients (*p* = 0.64), respectively, mostly Gustilo and Anderson type I and II. Midshaft femoral fracture were present in 51 (85%) patients in group A and 53 (85.5%) in group B (*p* = 0.93). Nine patients in both groups were admitted with distal shaft femoral fractures, representing 25% and 24.5% respectively (*p* = 0.93).

According to the AO classification, the more common type of fracture in our study was 32A3, with 17 patients in both groups representing 28.3% and 27.4% respectively (*p* = 0.91), followed by fractures type 32B2, with 12 (20%) patients in group A and 16 (25.8%) patients in group B (*p* = 0.44). All the fractures are presented in Table 2.

**Table 2:** Summary of fracture patterns

VARIABLE	STANDARD NAIL *n* = 60		FIN NAIL *n* = 62		*P*. value
	*n*	%	*n*	%	
Type of fracture according Gustilo and Anderson Classification:					
Closed	49	81.7	47	75.8	0.42
Open GI	5	8.3	7	11.3	0.58
Open GII	5	8.3	5	8.1	0.95
Open GIIIA	1	1.7	3	4.8	0.32
Type of fracture according AO classification:					
32A1	2	3.3	1	1.6	0.53
32A2	1	1.7	6	9.7	**0.05**
32A3	17	28.3	17	27.4	0.91
32B1	1	1.7	0	0	0.30
32B2	12	20	16	25.8	0.44
32B3	5	8.3	9	14.5	0.28
32C1	2	3.3	0	0	0.14
32C2	5	8.3	2	3.2	0.22
32C3	6	10	2	3.2	0.21
33A1	1	1.7	1	1.6	0.98
33A2	1	1.7	4	6.5	0.18
33A3	2	3.3	2	3.2	0.97
33C1	1	1.7	1	1.6	0.98
33C2	4	6.7	1	1.6	0.15

The mean delay between admission day and surgical intervention day was 9.3 (SD 14.5) days in group A and 9.6 (SD 13.7) days in group B (*p* = 0.90). The mean duration of surgical intervention was 104.3 (SD 42.2) minutes in group A and 78 (SD 32.4) minutes in group B (*p* =  < 0.001). Open reduction of the fracture was necessary in ten (16.7%) patients in group A and 12 (19.4%) patients in group B (*p* = 0.69).

At six months the loss of follow up was reflected with 17 (28.3%) patients treated with SSN® and 15 (24.2%) patients treated with SFN®. The most common complication was the presence of anterior knee pain during full weight bearing in three (5%) patients with SIGN® Standard Nail and eight (12.9%) with SIGN® Fin Nail (*p* = 0.12). Non-union at the six months post-surgical intervention was observed in one (1.7%) patient (AO 32B2) in group A and two (3.2%) patients in group B (AO 32C3 and AO 33C2) (*p* = 0.57).

Screw loosening was observed in one patient (AO 33A3) treated with SSN®, in patients treated with SFN® were no reported (*p* = 0.30). One patient in each group (32B3 and 32C2, respectively) developed migration of the nail (*p* = 0.98); We observed that all complications reported in the SFN group were patients with complex fracture patterns. Except for the anterior knee pain complication, all other patients with the above mentioned complications required a new surgical intervention. All complications reported in our study are described in Table 3.

**Table 3:** Summary of complications at six months of follow-up

VARIABLE	STANDARD NAIL *n* = 60		FIN NAIL *n* = 62		*P*. value
	*n*	%	*n*	%	
Screw Loose	1	1.7	0	0	0.30
Nail Breakage/Migration	1	1.7	1	1.6	0.98
Non-union	1	1.7	2	3.2	0.57
Anterior Knee Pain at Full Weight Bearing	3	5	8	12.9	0.12
Flexion Knee less than 90°	1	1.7	1	1.6	0.98
TOTAL OF COMPLICATIONS	7	11.7	12	19.4	0.24

## Discussion

**Fig. 4:**
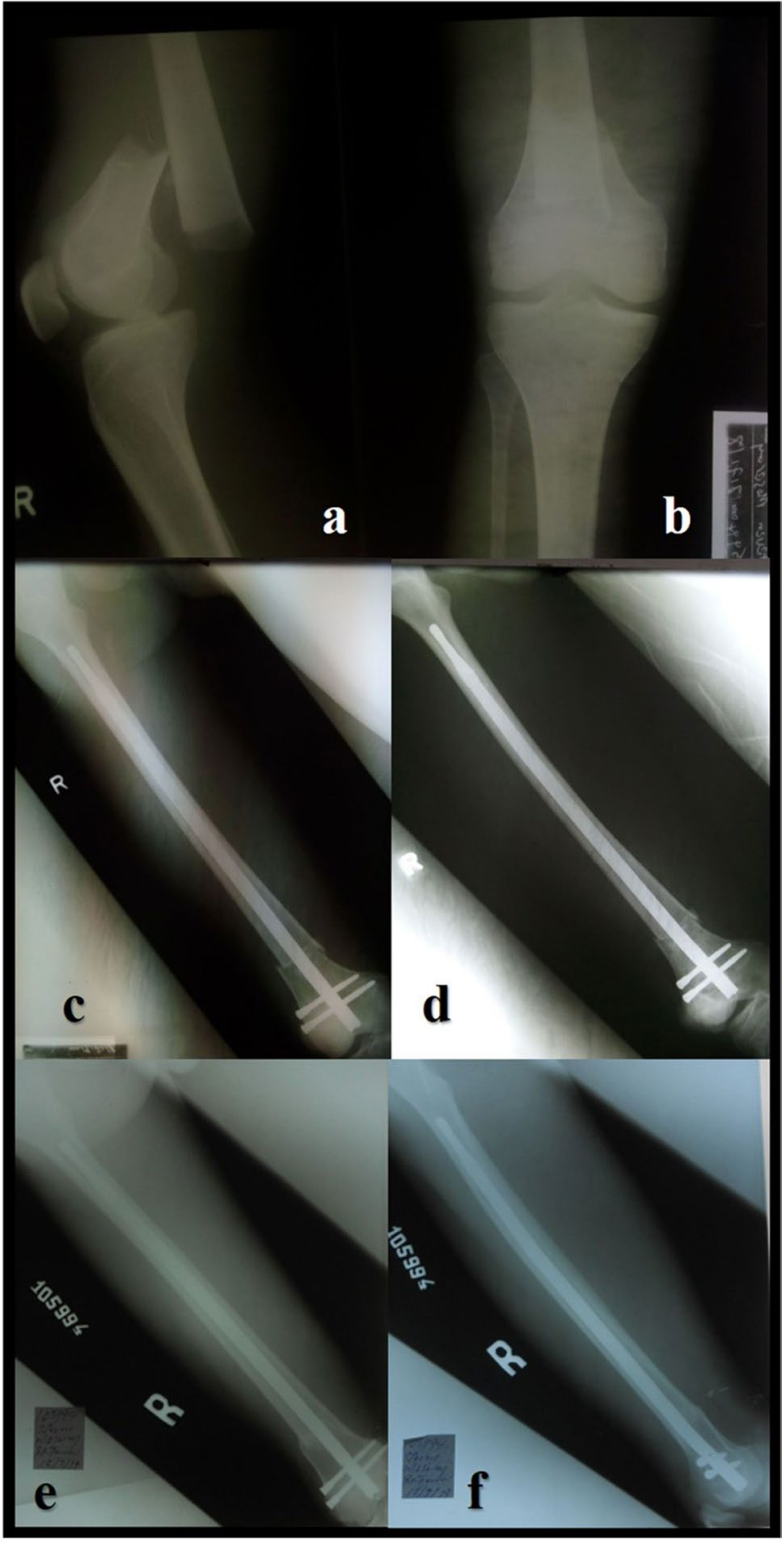
A 18 year old male patient with distal shaft fractures treated using SFN®, (**a**, **b**) preoperative radiographs, (**c**, **d**) postoperative radiographs, (**e**, **f**) consolidation at the follow up at 12 months

Intramedullary nailing (IMN) is a definitive femoral midshaft fractures surgical management method [19]. The advantages of IMM include the preservation of the haematoma, periosteum and minimal manipulation of the soft tissues around the fracture site, so it does not interfere with the biological process of consolidation [20]. Retrograde femoral nails can be used in all three types of femoral midshaft fractures 32A, 32B and 32C, also in distal shaft fractures (Fig. 4), such as supracondylar types 33A [20, 21], and sometimes in intercondylar type 33C of the AO classification could be used [21].

Retrograde IMM indications are polytraumatized patients with bilateral femoral fractures or ipsilateral fractures of the femur and tibia, distal shaft fractures, obesity with femoral midshaft fractures, and pregnancy [22]. In femoral fractures, the use of IMN with image intensifier is the standard technique. However, in LIMC where the image intensifier is not available in all scenarios, the locking targeting device in the SSN® is designed for use without an image intensifier or the availability of the SFN® that does not require distal locking screws is helpful in the treatment of long bone fractures in LIMCs [23].

Adesina et al. [24] compared 84 patients with antegrade nailing to 154 patients with retrograde nailing using the SIGN® program (SSN® and SFN®). They concluded that in low-resourced settings the retrograde nailing approach is more effective, patient positioning is easier, approaches are less complicated, and closed reduction and intramedullary reaming are more straightforward than antegrade nailing for the treatment of femoral midshaft fractures.

In our study, using the retrograde approach, we did not find a statistical difference between closed and open reductions of the fractures between the two nail options (*p* = 0.69); however, as the SFN® does not require locked screws, it decreases the number of surgical wounds and avoids distal screw loss or mistakes, which is a possible complication of SSN® when the image intensifier is not available.

Birner et al. [25] performed a randomized analysis of 500 patients treated at 110 hospital centers using retrograde femoral nailing with SFN® for femoral midshaft fractures, determining an incidence of malalignment > 5° in 9.4% of the cases and obtained satisfactory postoperative results in the majority of patients, comparable to use the SSN® in the treatment of femoral midshaft fractures in LIMC.

In Ethiopia, Birlie et al. [26] studied postoperative knee pain at six months in 110 patients treated with retrograde femoral nailing using the SSN® and SFN®. They reported that retrograde intramedullary nail fixation is an effective method for femoral midshaft fractures but may cause knee pain at six months in 36.4% of patients. In our study, at six months of follow-up, 9% (11 of 122) of the patients had anterior knee pain: 5% in group A and 12.9% in group B. No statistically significant differences were found between the two groups (*p* = 0.12).

Liu et al. [14] studied the outcomes of retrograde intramedullary nailing in 57 patients with SSN® and 28 patients with SFN® in Tanzania. At one year of follow-up, both surgical options offered similar radiographic, functional, and clinical results. Authors mentioned that the use of SFN® could decrease the surgical time. In our series, we confirmed that the mean operative time decreased from 104.3 min with SSN® to 78 min with SFN® (*p* =  < 0.001).

The principal limitation in our study was the loss of follow of the patients, generally in the NGO hospital is estimated that about 30% of the patients are lost to follow-up because they live in provinces far away from Freetown, there is no adequate transportation and many of the patients do not have the financial resources to return to the hospital. In many cases, not only patients treated with SIGN nail program, other patients with any pathology treated in the hospital only return for evaluation if they present complications. Based on this fact observed by the 23-year history of the hospital in Sierra Leone we could assume that patients with loss to follow-up at six months are without complications. Other limitations were the retrospective design of our study and the number of patients in the sub-groups, further studies with a larger number of patients would be needed to confirm our findings.

**Fig. 5:**
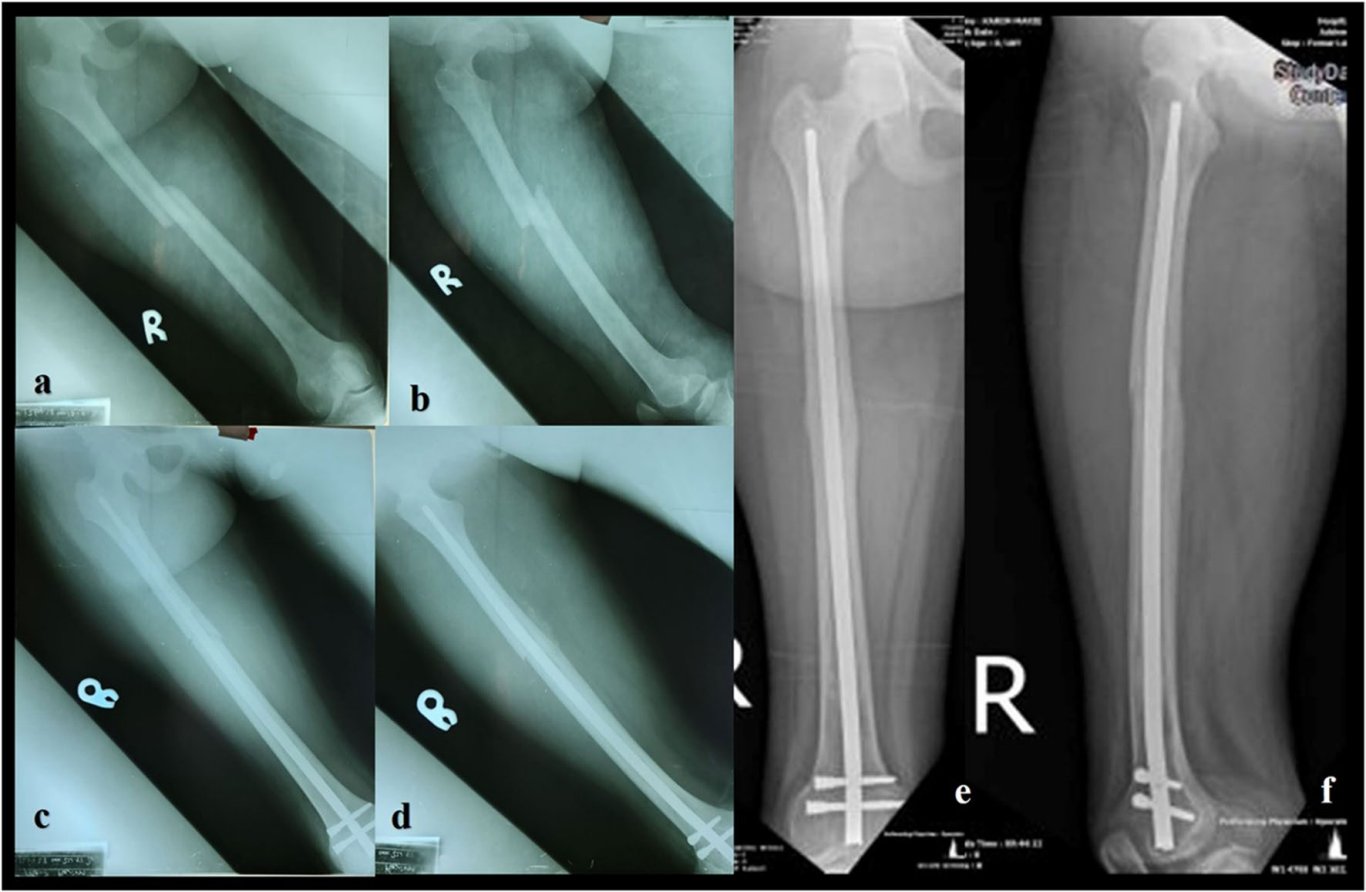
A 40 year old female patient with femoral midshaft fractures treated using SFN®, (**a**, **b**) preoperative radiographs, (**c**, **d**) postoperative radiographs, (**e**, **f**) consolidation at the follow up at 12 months

In conclusion, both options appear equally effective in the treatment of midshaft and distal femoral shaft fractures with excellent results. In our five-year experience using the sing nail program we observed that for patients with complex AO type fractures classifications like 32C2, 32C3, and 33C2 the SSN® offers better stability by using proximal and distal locking screws, in our study complications such as non-union and nail migration reported in the SFN group were patients with complex fracture patterns (32C2, 32C3, and 33C2). In other patterns of fractures, the SFN® is an excellent option (Fig. 5); The SFN® reduces the surgical time, due to this fact, in polytraumatized patients, patients with bilateral femur fracture or patients with ipsilateral tibia fracture, it can be considered as the best option to be used.

## Data Availability

Patient statistical data, images, tables, and any other information could be available by contacting by email drjperdomo@gmail.com, permission will be processed through the Emergency NGO to obtain the final authorization to share the information as they own the data rights.
